# Relationship between insulin resistance and subclinical atherosclerosis in individuals with and without type 2 diabetes mellitus

**DOI:** 10.1186/s40200-016-0263-5

**Published:** 2016-10-01

**Authors:** Hossein Fakhrzadeh, Farshad Sharifi, Mahtab Alizadeh, Seyed Masoud Arzaghi, Yaser Tajallizade-Khoob, Ali Tootee, Sudabeh Alatab, Mojde Mirarefin, Zohre Badamchizade, Hadi Kazemi

**Affiliations:** 1 Elderly Health Research Center, Endocrinology and Metabolism Population Sciences Institute, Tehran University of Medical Sciences, Fourth Floor, No 4th, Ostad Nejatollahi Street, Enghelab Avenue, Tehran, 15996615 Iran; 2 Endocrinology and Metabolism Research Center, Tehran University of Medical Sciences, Tehran, Iran; 3 Pediatrics, Shahed University, Faculty of Medicine, Tehran, Iran

**Keywords:** Coronary artery calcium score (CACS), Flow mediated dilation (FMD), Diabetes mellitus, Insulin resistance, HOMA-IR, Atherosclerosis, Cardiovascular diseases

## Abstract

**Background:**

Insulin resistance is of utmost importance as an underlying mechanism for increased risk of cardiovascular disease (CVD). We assessed the association between Homeostatic Model Assessment (HOMA-IR) and two surrogate subclinical atherosclerosis markers (SCA) among individuals with and without type 2 diabetes (DM), those who did not have any clinical presentation of the CVD.

**Methods:**

In a cross-sectional study, 208 participants (105 diabetics and 103 non-diabetics) were enrolled from referred patients with diabetes to an academic outpatient clinic and their non-diabetic relatives in-law. Fasting serum levels of insulin, blood glucose and lipid profile, were measured. Anthropometric and blood pressure were measuremented standardly. Body Mass Index (BMI) and Homeostatic Model Assessment-Insulin Resistance (HOMA-IR) index were calculated. Coronary Artery Calcium Score(CACS) was measured using a Multi-Detctor CT scanner. Flow mediated dilation (FMD) was measured using bimode ultrasonography (with linear transducer 13,000 MHZ). Univariate and multivariate logistic regression models were used to evaluate the association between these SCA markers and HOMA index in adjusting models.

**Results:**

CACS and HOMA-IR were higher and FMD was lower in diabetic participants than non-diabetic ones (*P* < 0.01) In a stepwise logistic regression model, CACS and FMD were associated with HOMA-IR (odds ratio = 1.778; 95 % confidence interval (CI): 1.211–2.726 and odds ratio = 1.557; 95 % CI: 1.601–2.275, respectively) in non-diabetics but not among diabetic participants.

**Conclusions:**

CACS and FMD are related to insulin resistance among non-diabetic individuals, but we could not find this relationship among diabetic patients.

## Background

Cardiovascular disease (CVD) is the leading cause of mortality worldwide [1]. Atherogenesis initiates since early life stages [2] and cardiovascular risk factors have been present for many years before the clinical atherosclerosis becomes evident [3]. Diabetes mellitus is an important risk factor for atheroma formation and atherosclerosis progression [4].

Insulin resistance and the ensuing hyperinsulinemia/hyperglycemia are critically important in increasing CVD risk [5]. In fact insulin resistance is a crucial link between diabetes and CVD [6]. Systemic insulin resistance produces a proatherogenic lipid phenotype by increasing very-low-density lipoprotein (VLDL) particles, which are metabolized to remnant lipoproteins promoting atherogenesis. Pro-inflammatory and pro-coagulant states which are induced by insulin resistance, also play important roles in atherogenesis [7].

It is well established that patients with type 2 diabetes have a higher prevalence of atherosclerosis as assessed by carotid intima-media thickness [8]. Coronary artery calcium score (CACS) is another surrogate atherosclerosis marker [9] which predicts coronary heart disease (CHD) in both non-diabetics [10] and diabetics [11], by visualizing coronary artery calcium. It is demonstrated that CACS has an added value to the established criteria of CHD identification [12]. Patients with a CACS ≥10 are at higher risk of CHD than those with CACS < 10 [13]. The results of the Framingham Offspring Study revealed that subjects with insulin resistance and type 2 diabetes had an increased burden of atherosclerosis as evidenced by higher CACS [14]. On the other hand, data have indicated that alterations in endothelial function may be followed by morphological changes that could contribute to atherosclerosis progression [15]. Endothelial dysfunction, proceeds by reduction of endothelium-derived nitric oxide. Physical stimuli such as shear stress and hypoxia could release nitric oxide from the endothelial cells [16]. Flow mediated dilation (FMD) is a method for measurement of the severity of vasodilation in response to shear stress. FMD is usually measured by computing the percent increase of brachial artery diameter after inducing shear stress by closing a calf around the arm [17].

Homeostatic model assessment (HOMA) index, is widely used for the assessment of insulin resistance as a valid surrogate of the gold standard method hyperinsulinemic-euglycemic clamp [18]. While not yet recommended for risk stratification, it is demonstrated that HOMA index is a reliable tool for prediction of the risk of coronary events [19]. It is also valuable for risk stratification in patients with angographically documented CHD [20].

We aimed to evaluate the association between two surrogate markers of atherosclerosis (CACS and FMD) with HOMA index in non-diabetic and diabetic individuals. We enroled both diabetic and non-diabetic subjects because we thought that the effective factors affect subclinical atherosclerosis may be differnet.

## Methods

We enrolled 208 (105 diabetic and 103 non-diabic) individuals. The individual with diabetes was selected by a systematic random method based on the patient ID code of the eligible individuals. The diabetic participants were recruited randomly from 20–70 year old patients with type 2 diabetes, who referred between December 2011 till Augst 2012, to Dr. Shariati University hospital Diabetes Clinic. The non-diabetic subjects were selected from non-bloody relations of diabetic participants (such as their hausbands or wives), who did not have diabetes in past medical history or in evaluations. The The inclusion criteria of the study necessitated that the participants be free of CVD or its symptoms. In addition none of the participants had an abnormal electrocardiogram at the time of recruitment or history of diabetes complications, as well (no sign of lower limb ischemia, retinopathy, nephropathy and neuropathy). The non-diabetic group were the relatives in-law of the diabetic participants. Those having at least two fasting glucose levels ≥126 mg/dl or using hypoglycemic agents, were considered as diabetic [21]. Sex, age, and history of consuming anti-hypertensive drugs were documented in a questionnaire filled out by a project assistant. Body mass index (BMI) was calculated after weight and height were measured using a calibrated electronic balance close to 0.1 Kg and a flexible tape to the accuracy of 0.5 cm. The measurements were performed in light clothing without shoes according to the standard methods. Smoking was defined as smoking any number of cigarettes in the last month prior to the study. After 15 min of rest in the sitting position, blood pressure was recorded twice using an automatic sphygmomanometer (Omron 7, Japan) based on the Seventh Report of the Joint National Committee on Prevention, Detection, Evaluation, and Treatment of High Blood Pressure (JNC7). Hypertension was defined by a mean systolic blood pressure ≥ 140 mmHg and/or a mean diastolic blood pressure ≥ 90 mmHg or consuming anti hypertensive medications [22].

Lower limb ischemia, retinopathy, nephropathy and neuropathy were ruled out by having an ankle brachial index (ABI) between 0.91–1.30, normal retinoscopy, albumin to creatine ratio < 30 mg/g in random spot urine sample or glomerular filtration rate (GFR) > 90 ml/min/1.73 m^2^ and normal monofilament 10 gr/diapason 250 Hz tests, respectively.

Using the photometry method (Pars Azmoon kits, Iran/auto analyzer Hitachi 902, Japan), a complete lipid profile was determined. Serum insulin levels were measured by radioimmunoassay method.

HOMA index was calculated using the following formula [23]:

$$ HOMA\  index = \Big[ plasma\  insulin\ \left(mIU/L\right) \times \left( plasma\  glucose\ \left( mg/ dl\right)\right]\ /\  405 $$

### Coronary Artery Calcium Score (CACS) measurement

Anterior-posterior and lateral chest scout views were obtained. Calcium score images were obtained with a Phillips 64 MDCT scanner using 64 × 2.5 mm × 400 ms with 120 KVP and 50–75 mAs to cover the entire heart and proximal ascending aorta. A positive calcium score was defined by 130 HU (Hounsfield units) with an area of ≥1 mm^2.^ The amount of calcium was quantified using the Agatston scoring method [24].

### Brachial Flow Mediated Dilation (FMD) measurement

We calculated the brachial artery FMD according to the American College of Cardiology guidelines [25]. We detected the edge of the borders of the brachial artery by a software installed on a high resolution ultrasonography device using a linear transducer 13,000 MHZ (MyLab 70 XVision, Biosound Esaote, USA). The measurement was conducted on right brachial artery 3–5 cm above the antecubital fossa just before cuff inflating and 60 s after cuff release. A cuff was inflated at least 50 mmHg above the systolic blood pressure around the right arm above the antcubial fossa for 5 min to produce a transient ischemia. The brachial FMD was calculated as percentage of alteration in brachial artery diameters induced by the shearing stress. All the measurements were performed in the end-diastolic phase coinciding with the R-wave of electrocardiogram. The average of the measurements during three consecutive cardiac cycles were considered as the final FMD score.

### Statistical analysis

SPSS software version 17.00 (SPSS Inc., Chicago, IL) was used for statistical analysis and *P*-values < 0.05 were considered significant. After performing Kolmogorov-Smirnov test to check the normality of the variables, we found that the HOMA index was not normally distributed. The participants were categorized according to CACS into two categories: CACS ≥ 10 and CACS < 10. The student *t*-test and Mann–Whitney *U*-test were used to compare the values between two groups. Both individuals with and without diabetes were classified into two groups based on the first FMD quartile and other participants. Skewed variables were reported as median (range) and normally distributed variables as mean and standard deviation (SD). Categorical variables were compared by *χ* ^2^ test. Univariabe binary logistic regression model was used to demonstrate association between the categorized CACS and FMD with HOMA index. We entered HOMA index, age, sex, BMI, waist circumference, HDL-C, hypertension and dyslipidemia, as predicting factors in a multivariable binary logistic regression using backward stepwise method (removed at 0.20 levels). Finally, for the categories of CACS in non-diabetic group adjustment was performed for age and dyslipidemia and in diabetic group correction was conducted for only age. As well, for the classified FMD, in non-diabetic group, the results were modified for age, sex, BMI, Waist circumference, and HDL-C. While in diabetic subjects this adjustment was carried out for age, BMI, waist circumference, and HDL-C.

### Ethical considerations

This study was approved in the endocrinology and metabolism research Institute ethical committee. (Registration code: 179/04/18/11). All stages of the study were conducted according to the Helsinki declaration principles. The participants signed the consent form after hearing the explanation about the objectives and the methods of the project by an experienced nurse. The confidentiality of data was heeded at all stages of the study.

## Results

A total of about 1500 diabetic cases referred to the diabetes clinic of Dr. Shariati hospital during the study period, of whom 428 subjects were eligible. Of these, 294 individuals refrained to participate and 28 subjects did not finish the data gathering process. Finally, 105 diabetic participants were enrolled. Also, we enrolled 103 non-diabetic individuals from in-law relatives of the diabetic participants as controls. The demographic characteristics of the participants is shown in Table 1.

**Table 1:** General Characteristics of the participants in two groups with and without diabetes

	The participants with diabetes	The participants without diabetes
	*N* = 105	*N* = 103
Age year (mean ± SD)	54.06 ± 8.24	49.73 ± 6.77
Sex female	51.4 %	55.3 %
Smoking	9.5 %	1.0 %
Body Mass Index kg/m^2^ (mean ± SD)	27.65 ± 4.18	28.18 ± 4.45
Waist Circumference cm (mean ± SD)	94.02 ± 10.71	92.44 ± 10.28
Hypertension	50.5 %	29.1 %
Low density Lipoprotein mg/dl (mean ± SD)	95.96 ± 25.17	113.27 ± 22.09
High density Lipoprotein mg/dl (mean ± SD)	41.22 ± 9.42	46.06 ± 11.09
Fasting Plasma Glucose mg/dl (mean ± SD)	166.46 ± 62.30	95.72 ± 11.60
Insulin IU/L (mean ± SD)	9.12 ± 13.34	8.79 ± 6.12
HOMA Index (mean ± SD)	4.01 ± 7.77	2.10 ± 1.52
Flow Mediated Dilation % (mean ± SD)	12.74 ± 6.76^a^	17.23 ± 10.05^b^
Coronary Artery Calcium Score	118.58 ± 380.90	32.78 ± 108.39

*SD* standard deviation, HOMA

^a^ *N* = 89

^b^ *N* = 94

More people in diabetic group than non diabetic group had CACS ≥10 than in non-diabetics (37.1 %, *n* = 39 vs. 17.5 %, *n* = 18, respectively; *P* < 0.01). The values of CACS were compared between two groups. There was a significant difference between the values of CACS among the diabetic and non diabetic groups (*P* < 0.01). Additionally, there was a significant difference between diabetic and non-diabetic participants in terms of FMD percents and HOMA index values (*P* values < 0.01 for both). We found a significant positive correlation between the HOMA index and BMI, fasting blood sugar (FBS), Triglyceride (TG), total cholesterol, low-density lipoprotein- cholesterol (LDL-C) and age in the diabetic participants. However, we did not observe a significant correlation between HOMA-IR and calcium score in individuals with diabetes and also in those who were non-diabetic.

After categorizing patients according to CACS into high risk and low risk groups for developing future CAD (CACS ≥10 and CACS <10), in diabetic and non-diabetic participants CACS was not significantly associated with HOMA index in a univariate logistic regression model, but it related to age in both groups. In multivariable logistic model categorized CACS was related to the HOMA index after full adjustments in non-diabetic group (odds ratio = 1.778; confidence interval 95 % 1.211–2.736) but there was no association between CACS and HOMA- IR in diabetics (Table 2). Furthermore the categorized FMD had no relationship with HOMA- IR in uniivarble model; While it was associated with HOMA index after adjustment for potential confounders (odds ratio = 1.557; confidence interval 95 % 1.066–2.273) in non-diabetic group (Table 3).

**Table 2:** Association between coronary artery calcium score categorized (≥10 and <10) with HOMA Index in univariate and multivariable logistic regression model^a^

	Univariabe model		Multivariable model	
	Participants with diabetes	Participants without diabetes	Participants with diabetes	Participants without diabetes
	Odds ratio (CI 95 % odds ratio)	Odds ratio (CI 95 % odds ratio)	Odds ratio (CI 95 % odds ratio)	Odds ratio (CI 95 % odds ratio)
HOMA	0.866 (0.717–1.046)	1.253(0.930–16.689)	0.960 (0.783–1.177)	1.778* (1.211–2.736)
Age	1.16* (1.083–1.238)	1.212* (1.097–1.339)	1.153* (1.076–1.236)	1.313* (1.147–1.501)
Sex (female)	0.606 (0.273–1.345)	0.588 (0.211–1.637)	-	-
BMI	0.976 (0.886–1.075)	1.062 (0.951–1.186)	-	-
6WC	0.983 (0.947–1.021)	1.096 (1.031–1.165)	-	-
HDL-C	1.006 (0.965–1.049)	1.023 (0.978–1.070)	-	-
Hypertension	2.041 (0.910–4.578)	2.291 (0.803–6.539)	-	-
Dyslipidemia	0.671 (0.295–1.526)	2.146 (0.737–6.247)	-	4.323 (0.983–19.018)

Independent variables were Age, Sex, BMI, Waist Circumference, LDL-C, HDL-C, Hypertension, Hyperlipidemia

*CI* confidence interval, *BMI* body mass index, *WC* waist circumference, *HDL-C* high density lipoprotein cholesterol

*P < 0.05

^a^ Backward Conditional Multivariable Logistic Regression Model (probability for stepwise: entry: 0.05 removal: 0.20)

**Table 3:** Association between Flow Mediated Dilation quartile (first and other quartiles) with HOMA Index in univariate and multivariable logistic regression model^a^

	Univariabe Model		Multivariable Model	
	Participants with diabetes	Participants without diabetes	Participants with diabetes	Participants without diabetes
	Odds Ratio (CI 95 % odds ratio)	Odds Ratio (CI 95 % odds ratio)	Odds Ratio (CI 95 % odds ratio)	Odds Ratio (CI 95 % odds ratio)
HOMA	0.978 (0.887–1.078)	1.177 (0.884–1.566)	0.973 (0.836–1.132)	1.557* (1.066–2.275)
Age	1.038 (0.980–1.101)	1.084* (1.002–1.172)	1.058 (0.990–1.132)	1.137* (1.021–1.265)
Sex (female)	0.859 (0.327–2.256)	0.204 (0.071–0.582)	-	0.033 (0.004–0.310)
BMI	1.065 (0.952–1.191)	1.037 (0.935–1.150)	1.241 (0.997–1.546)	1.642* (1.179–2.289)
WC	0.977 (0.935–1.020)	1.027 (0.980–1.077)	0.925 (0.841–1.017)	0.817* (0.706–0.946)
HDL-C	0.937* (0.881–0.997)	0.919* (0.866–0.975)	0.912* (0.850–0.978)	0.905* (0.838–0.979)
Hypertension	2.208 (0.799–6.104)	2.265 (0.848–6.051)	-	-
Hyperlipidemia	1.394 (0.480–4.044)	0.596 (0.231–1.539)	-	-

Independent variables were Age, Sex, BMI, Waist Circumference, LDL-C, HDL-C, Hypertension, Hyperlipidemia

*CI* confidence interval, *BMI* body mass index, *WC* waist circumference, *HDL-C* high density lipoprotein cholesterol

*P < 0.05

^a^Backward Conditional Multivariable Logistic Regression Model (probability for stepwise: entry: 0.05 removal: 0.20)

## Discussion

We found that CACS was related to HOMA-IR in non-diabetics. By each unit increase of HOMA-IR, we found more than 75 % increased risk of high CACS. This finding has a good concordance with the results of several studies which notified a significant association between insulin resistance and CVD risk. For example Bertoluci et al. showed that in patients with angiographic CHD, a HOMA index **>** 6.0 was associated with life-threatening coronary lesions and suggested the HOMA index as an independent predictor of CHD [26]. In another study in non-diabeticss, HOMA over 1.80 for women and 2.12 for men was demonstrated to be predictive of increased risk of cardiovascular events and mortality [27]. This relation has also been observed in longitudinal population studies [15]. The Framingham offspring researchers found a clear pattern of an increasing burden of subclinical coronary atherosclerosis evidenced by higher CACS, with increasing severity of glucose intolerance [15]. Lee et al. in a 24 month study of 869 healthy adults aged 60 to 72 years who were free of clinical CHD at enrollment, showed that Insulin resistance independently predicted progression of CACS [28]. Also in an older cohort, insulin resistance has been suggested to increase CACS prevalence and progression in both men and women [25]. Also a significant association between advanced left anterior descending coronary artery calcium deposition and glucose intolerance (HBA_1_C ≥ 8.0). has been observed in the autopsy findings of young adults [29]. Recently Sung et. al in a large cohort of 10,511 young adult Korean men and women at low risk for CVD found that insulin resistance was an independent predictor of high CACS in men (*P* = 0.03); with more impact on CVD risk and CACS in young men compared with women. In this cohort diabetes was also significantly associated with high CACS in men. Men with diabetes had 3.8-fold increase in CACS prevalence compared with insulin sensitive men without diabetes. They suggested that individuals with early CACS deposition are likely at the highest risk for future CHD compared with their cohorts with low CACS [23].

On the other hand, we found that an increase in HOMA-IR, was related to disturbance in FMD in the nondiabetic participants. There are enormous clinical and experimental studies which stipulate that insulin resistance might be a fundamental underlying metabolic disturbance in CVD developmente. Furthermore, endothelial dysfunction is one of the main components of of insulin resistance [30]. Suzuki et al. in a study found that insulin resistance was the sole predictor of FMD disturbance [26].

We could not find any relationship between CACS and FMD with insulin resistance among the diabetics. There exists justifications for this apparent inconsistency. First, estimation of insulin resistance based on HOMA-IR in diabetic patients may be distorted due to taking exogenous insulin with consequent development of pseudohyperinsulinemia. Particularly, we did not measure C-peptide [31]. Another explanation could be that CACS and FMD in diabetic individuals are related to several other factors; such as glycemic control, diabetes duration, diabetes complications, genetic factors, etc. which we were not able to include in our calculations, mainly due to limited sample size. These factors potentially confound the relationship between insulin resistance and surrogate atherosclerosis markers.

In general chronic subclinical inflammation due to insulin resistance is potentially responsible for increased CVD risk in those with hyperinsulinemia [32]. In animal models insulin has been shown to enhance the proliferation of smooth muscle cells [33]. Chronic exposure to advanced glycated end products with ensuing chronic subclinical inflammatory signaling also plays important roles in development of atherosclerosis in type 2 diabetics [34].

We used the cut off point of 10 (instead of 100) for categorization of CACS; as there are studies which report the cutoff of 10 to be more sensitive, albeit less specific for prediction of atherosclerosis [22]. Another reason was that the number of non-diabetic individuals with CACS ≥ 100 was few.

We also observed that the surrogate atherosclerosis markers were related to age in both diabetic and non diabetic participants. This finding was in concordance with results of other studies which reported an association between CAD and age [35].

Our finding of increased incidence of high CACS and FMD in asymptomatic diabetic subjects relative to controls was concordant with results of Schurgin et al. who also showed an increased prevalence (26 %) of high CACS in patients with diabetes compared to controls (14 %, *P* < 0.004) [35]. Wagenknecht et al. also found an increased prevalence (27 %) of high CACS in probands of type 2 diabetics compared with nondiabetic siblings (8 %, *P* < 0.003) [36]. Lately, researchers in the large Multi-Ethnic Study of Atherosclerosis cohort showed that individuals with MetS and DM have a greater incidence and absolute progression of high CACS compared with individuals without these conditions, with progression also predicting CHD events in those with MetS and DM [37].

To our knowledge this is the first study in Iran, which studies CACS and FMD as markers of atherosclerosis among diabetic patients.

This study had limitations. First, this was a hospital-based study with constraints to extend the results to the population. Second, the sample size was not ideally large which might bear some bias inherently as we were not able to enter some other confounding factors such as duration of diabetes in our model. In addition this was a cross-sectional study and causality interference is not possible for these types of studies. On the other hand, there are other documented markers of atherosclerosis such as carotid intima-media thickness (CIMT) which have been demonstrated to be of value in the assessment of the potential risk for future cardiovascular events [38]. The other weak point is a relatively wide variance in the duration of diabetes among our diabetic group which could interfere as a confounder in our investigation. We suggest future studies with inclusion of other surrogate atherosclerosis markers such as CIMT, and with larger sample size to shed more light on the potential relationship between insulin resistance and subclinical atherosclerosis.

## Conclusion

Our study showed that the markers of subclinical atherosclerosis such as CACS and FMD were related to insulin resistance as indicated by increased HOMA index among non-diabetic subjects but not in diabetics. This finding could underline the role of insulin resistance in developing the complications of atherosclerosis in non-diabetic subjects. At the end, our observations imply that measurement of serum insulin and calculation of the HOMA index even in nondiabetic individuals may have an added value to predict atherosclerosis over the next years.

## Abbreviations

ABI: Ankle brachial index
BMI: Body mass index
BS: Bachelor of Sciences
CACS: Coronary artery calcium score
CHD: Coronary heart disease
CI: Confidence interval
CIMT: Carotid Intima-media thickness
CVD: Cardiovascular disease
DM: Diabetes mellitus
FMD: Flow mediated dilation
GFR: Glomerular filtration rate
HBA1C: Hemoglobin A1C
HDL-C: High density lipoprotein cholesterol
HOMA-IR: Homeostatic model assessment-insulin resistance
JNC7: Seventh Report of the Joint National Committee on Prevention, detection, evaluation, and treatment of high blood pressure
LDL-C: Low density lipoprotein cholesterol
MD: Medical doctor
MDCT: Multiple detector computed tomography
MetS: Metabolic syndrome
MHZ: Mega Hertz
SCA: Subclinical atherosclerosis
SD: Standard deviation
TG: Triglyceride
VLDL: Very-low-density lipoprotein
